# “There’s a lot of stereotypes going on”: A cross-national qualitative analysis of the place of gender in declining youth drinking

**DOI:** 10.1016/j.drugpo.2022.103827

**Published:** 2022-08-16

**Authors:** Amy Pennay, Jukka Törrönen, Maria Dich Herold, Laura Fenton, Sarah MacLean, Gabriel Caluzzi, Hannah Fairbrother, Vibeke A. Frank, Eva Samuelsson, John Holmes

**Affiliations:** a Centre for Alcohol Policy Research, La Trobe University, Melbourne, Australia; b Department of Public Health Sciences, Stockholm University, Sweden; c Centre for Alcohol and Drug Research, Aarhus University, Aarhus, Denmark; d School of Health and Related Research, University of Sheffield, Sheffield, UK; e School of Allied Health, Human Services and Sport, La Trobe University, Melbourne, Australia; f Health Sciences School, University of Sheffield, Sheffield, UK; g Department of Social Work, Stockholm University, Sweden

**Keywords:** Alcohol, Non-drinking, Young people, Gender, Cross-national

## Abstract

**Introduction:**

Significant declines in drinking among young people have been recorded in many high-income countries over the past 20 years. This analysis explored the role of gender - which we interpret as socially constructed and relational - to provide insight into whether and how gender might be implicated in declining youth drinking.

**Methods:**

Interview data from four independent qualitative studies from Australia, Denmark, Sweden and the UK (n=194; participants aged 15-19 years) were analysed by researchers in each country following agreement about analytical focus. Findings were collated by the lead author in a process of ‘qualitative synthesis’ which involved successive rounds of data synthesis and feedback from the broader research team.

**Findings:**

Our analysis raised two notable points in relation to the role of gender in declining youth drinking. The first concerned the consistency and vehemence across three of the countries at which drinkers and states of intoxication were pejoratively described in gendered terms (e.g., bitchy, sleazy). The second related to the opportunities non- and light-drinking offered for expressing alternate and desirable configurations of femininities and masculinities.

**Conclusions:**

We identified an intolerance towards regressive constructions of gender that emphasise weakness for women and strength for men and a valorisation of gendered expressions of maturity through controlled drinking. Though subtle differences in gendered drinking practices between and within countries were observed, our findings offer insight into how young people’s enactions of gender are embedded in, and evolve alongside, these large declines in youth drinking.

## Introduction

There is a long history of studying gender in alcohol research, both in terms of investigating differences in the way women and men drink (e.g., Wilsnack, Wilsnack, Kristjanson, Vogeltanz-Holm, & Gmel, 2009), but more importantly for our purposes, in the way drinking, and particularly intoxication, is shaped by and expressed through socially constructed ideas about gender (e.g., Jackson & Tinker, 2007; Mullen, Watson, & Black, 2007). In recent years, researchers have highlighted how traditional constructions of gender continue to shape drinking practices among young adults (Frank, Herold, Schrøder, Bjønness, & Hunt, 2020; MacLean, Pennay, & Room, 2018; Nicholls, 2020), and conversely, an emerging convergence in expectations and practices along gender lines (e.g., Bogren, 2020; Törrönen & Roumeliotis, 2014).

Alongside these progressive developments in the theorisation of gender and drinking, significant declines in drinking among young people have been recorded over the past 20 years in most high-income countries, particularly for teenagers (Vashishtha et al., 2021). We have argued that to contextualise the widespread nature of these changes, we must adopt a cross-national lens (Pennay et al., 2018). Members of our research team have also suggested that the shifting nature of gender identities may be playing a role in the diminishing cultural position of alcohol for young men and women, but in different ways (Kraus et al., 2020). In this article, we attend to these propositions and examine the role of gender in expressions of drinking among young people at a time when alcohol use is declining. We take a cross-national approach to explore expressions of gender and drinking in four high-income countries where declining drinking among young people has been observed over the past two decades: Australia, Denmark, Sweden and the UK.

### Gender and alcohol

Broadly speaking, we understand gender as socially constructed, performed and relational. Gender is defined by the World Health Organisation as ‘the characteristics of women, men, girls and boys that are socially constructed. This includes norms, behaviours and roles associated with being a woman, man, girl or boy, as well as relationships with each other. As a social construct, gender varies from society to society and can change over time’ (World Health Organisation, 2022). We understand gender to be a ‘doing’, constituted by the performativity of the everyday and reproduced through the (repetitive) practices of what is deemed ‘appropriate’ or ‘typical’ for men and women (Butler, 1990; West & Zimmerman, 1987). In this respect, we understand gender performances as both conscious and unconscious, and often taken for granted (see also Hennell, Limmer, & Piacentini, 2022).

In 2002, Measham claimed that ‘doing drugs is doing gender’. She suggested that for women, ‘doing gender’ through ‘doing drugs’ enabled the possibility of both reproducing and challenging traditional and non-traditional femininities (Measham, 2002). Work in ensuing years has explored the complex and dilemmatic ways young women use alcohol and perform intoxication, both reifying and contesting what are considered ‘appropriate femininities’ (Griffin, Szmigin, Bengry-Howell, Hackley, & Mistral, 2013; Hutton, 2020). Similarly, drinking and intoxicated practices for young men have been constructed on the one hand as productively affirming masculinity, bonding and solidarity, and on the other, problematised and pathologised as expressions of hegemony or aggression (Hunt & Antin, 2019; O’Brien et al., 2018; Wilkinson & Wilkinson, 2020).

Of course, these binaries are at the extreme ends, with contemporary social science research on gender and alcohol more commonly emphasising convergent, contingent and fluid masculinities and femininities of drinking. For example, Demant and Törrönen (2011) proposed that a ‘tendency toward feminisation’ has transformed the drinking cultures of young men and women in Denmark and Finland into more ‘fragmented drinking cultures’, thereby producing ‘a variety of possible feminine and masculine positions in drinking’ (pg. 1244). In particular, scholars have drawn attention to the importance of contextual and social factors in shaping gendered expressions, such as drinking location (Demant & Törrönen, 2011; Törrönen & Maunu, 2007), place (Herold, Hunt, & Antin, 2020; MacLean & Moore, 2014; Törrönen, Rolando, & Beccaria, 2017), and class (Bogren, 2020 Törrönen & Roumeliotis, 2014; Vaadal & Dahl, 2021).

A recent special section in the International Journal of Drug Policy was dedicated to ‘Young People, Gender and Alcohol Intoxication’ (2020, volume 81). While we cannot detail all the rich insights provided in this issue, two salient points are worth noting. First, it was apparent from research across many high-income countries that gender, whether it was reifying, contesting or reshaping dominant understandings of masculinities and femininities, was ‘performed at great lengths, both corporally and discursively’ (Herold & Hunt, 2020, pg. 8; see also Nicholls, 2020; Wilkinson & Wilkinson, 2020). Second, gender was often made most salient through stereotyping and stigmatising processes, for example, when participants distanced themselves from the drinking of particular ‘types’ of young men or women (Bogren, 2020; MacLean, Demant, & Room, 2020; Wilkinson & Wilkinson, 2020).

### Gender and non-drinking

It has been suggested that the heavier the drinking situation, the more visible gender tensions become (Demant & Törrönen, 2011; Törrönen & Maunu, 2007). This raises the question of how masculinities and femininities are expressed through non- or light-drinking practices. The role of gender and non- or light-drinking among young people has been less explored until recently (Frank et al., 2020; Törrönen, Samuelsson, & Roumeliotis, 2020; Törrönen, Samuelsson, Roumeliotis, Room, & Kraus, 2021). This work has suggested that gendered norms may be influencing the cultural devaluation of drinking for young men and women but in different ways. In particular, it has been suggested that young men and women are expressing masculinity and femininity through divergent health practices. For example, in Sweden and Denmark, young men’s accounts suggested that they were performing masculinity through activities such as sport (Frank et al., 2020; Törrönen, Roumeliotis, Samuelsson, Kraus, & Room, 2019; Törrönen et al., 2020), and in this sense, masculinity emerged ‘as more flexible and less attached to heavy drinking’ (Törrönen et al., 2019, pg. 16). On the other hand, young women’s accounts emphasised healthy eating and wellness, vulnerability and responsible femininity, and a reluctance to challenge traditional feminine boundaries of acceptable drinking (Frank et al., 2020; Törrönen et al., 2019; Törrönen et al., 2020).

### Gender and alcohol in a cross-national frame

Cross-national analyses have long been, and continue to be, highly influential in alcohol research. For example, the typology of ‘wet’ and ‘dry’ drinking cultures, otherwise known as wine and beer cultures, or Mediterranean and Northern European drinking styles, emerged from analyses of cross-national drinking patterns (Room, 1988). Quantitative cross-national analyses have suggested a convergence in patterns of adolescent intoxication across many European countries, meaning young men no longer drink much more heavily than young women (Kuntsche et al., 2011). Qualitatively, cross-national analyses continue to be an insightful way of exploring the role of gender in shaping the meanings of drinking. For example, Törrönen and Roumeliotis (2014) emphasised how women’s independence had shaped drinking in Finland and Sweden into a ‘more diverse and heterogeneous phenomenon within and across gender groups’ but that they ‘var(ied) locally, regionally and globally, intersect(ed) in specific ways with class, age and generation, and form(ed) multidimensional, paradoxical and tension-driven relationships with each other and with femininities’ (pg. 126). Comparative work involving focus groups in Scandinavia and Italy also recently reported a diversification of gendered drinking accounts between the two regions according to drinking situations, age and education (Törrönen et al., 2017).

Cross national studies are important because they often challenge taken for granted assumptions around social processes that are rooted in the experience of particular countries (Chapple & Ziebland, 2018). They enable us to theorise about trends that occur across countries, to explore variation between countries, and in doing so can complicate or unsettle our expectations of complex social relationships (Baistow, 2000). Quantitative cross-national analyses have been useful for identifying changes in the magnitude and timing of recent trends in young people’s drinking across high income countries as well as the various factors influencing the declines (e.g., de Looze et al., 2015; Pape, Rossow, & Brunborg, 2018; Vashishtha et al., 2020; Vashishtha et al., 2021). However, there have been few, if any, qualitative cross-national studies of the decline in young people’s drinking.

Given the rich insights provided by cross-national studies, and the enduring, important role of gender in drinking practices, in this article we explore whether and how gender might be implicated in significant declines in drinking among young people. We do so through the exploration of qualitative data from four high-income countries observing these declines.

## Methods

The present analysis draws together findings from separate qualitative projects undertaken over similar time periods in four countries. The methods and samples of the studies differed, as did the focus of interview questions. It should be noted that the Australian study consisted entirely of non and light drinkers, the Danish study entirely of drinkers, and the Swedish and UK studies a mix of non, light and heavier drinkers. The Danish study was particularly focused on gender and drinking, the Australian and Swedish studies explored gender as a theme in interviews, and the UK data did not include any questions with an explicit focus on gender. Given this, our aim is not to directly compare differences between countries - though certainly to raise differences where they are apparent - but rather, to develop a broader understanding of the role of gender in drinking and non-drinking practices in high-income countries where young people’s alcohol consumption has declined.

### Samples and procedures

#### Australia

In 2018, 50 individual face-to-face interviews were under-taken with abstainers (n=25) and light drinkers (typically consuming fewer than four drinks on an occasion, monthly or less often, n=25) aged 16-19 years from Melbourne, Victoria. Participants were predominantly recruited through social media, with a small number via snow-balling or word of mouth. The sampling frame aimed for an equal split of gender and variety of locations (e.g., inner city and outer suburban, middle- and working-class areas) within driving distance for the Melbourne-based research team. Semi-structured, one-to-one interviews were undertaken in public spaces (cafés, parks, libraries, shopping malls, university campuses) or private homes; participants were reimbursed AUD$50 for participation. Signed informed consent was provided before interviews, which lasted approximately 60 minutes on average, and were recorded and transcribed verbatim (for further details see Caluzzi, MacLean, Livingston, & Pennay, 2021; Caluzzi, MacLean, & Pennay, 2020; Caluzzi, Pennay, & MacLean, 2021; Caluzzi, Pennay, MacLean, & Woodman, 2021).

#### Denmark

In 2015-2016, 140 face-to-face in-depth interviews were undertaken with young people between 18-25 years of age who had used alcohol within the past three months. The interviews formed the empirical basis of a large qualitative study on young people, intoxication and gender. For the present analysis, only participants aged 18-19 years were included (n=35) to ensure our sample ages were similar across the participating countries. Participants living in all regions of Denmark were recruited through street-level approaches, flyers, social media, snowballing and educational institutions. Interviews were undertaken in participants’ homes, educational institutions, library meeting rooms or university offices. Signed informed consent was provided before interviews, which generally lasted between 2-3 hours, were recorded and transcribed verbatim. All received a gift card for a movie theatre for their participation (for further details see Frank et al., 2020; Herold & Hunt, 2020; Jensen, Herold, Frank, & Hunt, 2019).

#### Sweden

In 2017 and 2018 interviews were conducted with 56 young people aged 15-19 years from different socioeconomic, ethnic, and geographical backgrounds in Sweden. Non-drinkers, moderate drinkers and heavy drinkers (drinking to intoxication at least once a month) were interviewed. Ten participants were interviewed with friends in pairs, and 46 participants were interviewed individually. Interviewees were recruited by purposive sampling from various secondary (9th grade) and upper secondary (12th grade) schools in the Stockholm region and other towns in the middle of Sweden. Contacts through non-governmental organisations and social media were used to recruit interviewees. Signed informed consent was provided before interviews, which generally lasted between 35 and 90 minutes. All received a gift card for a movie theatre for their participation. The interviews were recorded and transcribed verbatim (for further details see Törrönen et al., 2019; Törrönen et al., 2020; Törrönen et al., 2021).

#### United Kingdom

Friendship group and one-to-one interviews were undertaken with young people in 2018-19. A total of 53 young people aged 16-19 were interviewed. Younger and older cohorts were also interviewed but these data are not included in this analysis. For those aged 16-19, all but one was interviewed in a friendship group. Interviews used creative, participatory methods, including a relationship map and visual aids. In terms of drinking practices, 12 were abstainers, 10 were light drinkers (defined as typically drinking no more than once a month and rarely drinking more than 1-2 drinks), and 31 were drinkers (defined as typically drinking more often than once a month and drinking more than 2 drinks on a typical drinking occasion). All participants lived in or on the suburban or rural outskirts of Sheffield. They were recruited from socio-economically and geographically contrasting schools (two affluent, one deprived and two rural), two further education (FE) colleges, which offer academic and vocational qualifications to students aged 16 and over, and two universities. Interviews with school- or college-aged participants were conducted on school or college premises, while older participants were typically interviewed in public spaces. Signed informed consent was provided before interviews. Interviews lasted approximately 45-60 minutes on average, and were recorded and transcribed verbatim. Interviewees were offered shopping vouchers as reimbursement for their time.

Ethics approval for cross-national comparative analyses was provided by La Trobe University (HEC19479). The demographic characteristics of all participants are provided in Table 1.

### Analysis

Data and analyses were undertaken by researchers in each country to facilitate the production of culturally sensitive and meaningful accounts. Through a series of online meetings, researchers agreed on a conceptual framework and coding matrix before researchers independently analysed their respective datasets. The similarities in the methodologies of the projects to some extent eased analytical problems and allowed us to develop categories that were common to the various projects (Liamputtong, 2008). Based on our knowledge of our data as well as our readings on alcohol use and gender, the a priori coding matrix was simple, but allowed for inductive coding underneath the main codes:

- Expressions of gender concerning own drinking or non-drinking
- Expressions of gender concerning others’ drinking (i.e., stereotyping or stigmatising of drinkers or non-drinkers)
- Evidence of moderators or contingencies (i.e., place, cultural background, class, etc.) concerning gender and drinking

Researchers met online to discuss findings after the initial round of analyses and continued to provide feedback and debate others’ accounts as the production of findings progressed, constituting a process of ‘qualitative synthesis’ (Liamputtong, 2008; Troman & Jeffrey, 2007). Researchers from each country contributed quotes representative of themes to a table for ease of selection. Included quotations were ultimately selected by the lead author. Danish and Swedish quotations were translated to English by the lead researchers from those countries. All participants’ names have been replaced with pseudonyms.

Gender was both explicit and implicit in our data. Our exploration of gender included discursive representations of men, women, femininities and masculinities; affective expressions of gender; and socio-cultural expectations and norms relating to gender (e.g. Herold & Hunt, 2020; Törrönen et al., 2020). Our individual analyses of gender were likely shaped by each researcher’s disciplinary background (e.g., sociologists, public health researchers), epistemological (e.g., constructivist, critical realist) and reflexive (e.g., middle-class, Caucasian) positionings. We did not disagree at any stage of analysis, but often layers of nuance were added to the findings as presented when researchers felt something more complex was apparent in the data than first interpreted. The findings that follow are organised into our matrix structure, focusing on the gendered expressions of own (non)drinking, gendered expressions of others’ (non)drinking and the moderators of these.

## Findings

### Drinking and non-drinking as gendered performances

In all four countries, we identified that drinking and non-drinking practices continue to enable opportunities for traditionally constructed gendered expressions and performances. There were many similarities in how these expressions were enacted across the countries; for example, through beverage choice (e.g., wine for women and beer for men), gender normative performative practices (e.g., different styles of dancing and bonding) and gendered enactments of discipline and control. Consider, for example, these typical extracts from a young Australian woman and Danish man:

Like we get a bit silly and like by the end of the night, it was like 1am, and my friend and I were going to get an Uber home and she was acting drunk but she hadn’t had anything, like we’d only just had like had a few glasses of champagne … but like she – like I don’t know, the way that we were just like really happy and ditzy, I guess, it was kind of enough for us … I get delirious and like really happy when I’ve been dancing with friends or whatever, or like when you get dressed up for something, you just – it makes you feel a little bit better about yourself. (Jessie, 16yo, Australian)

We are loud. Very loud. In a good mood [as the group make their way from the pre-party to the party]. And earlier, before my girlfriend, it was like ‘we’re gonna pick up some ladies’ and we just have to do ‘this and that’. And we’re just happy, and up for it and young. Now, were more just going down there to have a cosy time together, drink some alcohol. (Emil, 19yo, Danish)

These excerpts show how young women and men incorporated gendered language into their descriptions of same-sex bonding. Such descriptions commonly shifted from more stereotypically feminine descriptions of giggling and dancing for women, to more typically masculine descriptions of ‘blowing off steam’ and ‘getting rowdy’ for men. Similar examples were evident from Swedish and UK participants.

Gender was also apparent in performative work related to the construction of non-drinking identities. For men, masculine identities were often described regarding sport and/or the desire for a strong physical appearance. Gaming too, was a common way of expressing masculinity as a non-drinker. For women, the range of non-drinking biographies were significantly more diverse. Health (e.g., looking slim or fit, feeling strong and powerful) and religion (e.g., performing care giving activities around church, dressing in feminine but ‘classy’ clothes for church), were some of the more common ways of expressing femininity but there were also many creative forms of doing gender articulated by women (e.g., around dance, photography, film). This theme was particularly strong in Australia and Sweden, where participants described careful attention to displays of various forms of masculinity or femininity on social media, for example:

I mean I like people to - I like posting [on social media] really nicely taken photos, and quite arty. So that and dance photos because I want people to know that I dance. Then also with friends because I want people to know that [just because I’m not partying] I’m not a loner, I want to show that I have friends and stuff. (Bella, 16yo, Australian)

In all countries, gender could be expressed through light drinking practices, or through consciously limiting drinking or ‘managing’ drunkenness, via descriptions of exercising discipline and control. Often these descriptions focused on maturity. For example, in the UK some men emphasised being able to ‘handle’ their drink (although this often still involved consuming substantial quantities of alcohol). In Australia, it was common for young men to describe drunkenness as something boys do, and resisting drunkenness as an accomplishment reserved for men. In all countries, young women spoke about ‘knowing their limits’. For some women, limiting drinking was positioned with respect to safety and vulnerability. For others, it was couched in terms of maintaining ‘appropriate femininities’. In these ways, light and moderate drinking practices also offered up ways of ‘doing gender’:

You have to look nice when you drink as a woman. I think that some guys think it’s cool when girls just pour it down. But it’s just not very feminine … I never just pour it down. I think it looks nicer when you drink more slowly, have a straw, so you don’t mess everything up. (Katrine, 19yo, Danish)

We also identified many examples where stereotypical notions of gender were contested through drinking performances and discussions about gender and drinking. For example, men noting a preference for sweet drinks, women reporting a dislike for feminine party attire, and women and men resisting displays of ‘appropriate femininities’ and masculine performances of alcohol use. For example:

My circle of friends has been very much like feminist-active … the girls do not care what men think [about their intoxication] and so on so it’s a good thing, they don’t care what people think of them. (Oliver, 18-19yo, Swedish)

The clear opposition towards traditional masculine drinking norms was one of the strongest findings to emerge from Australian data (bearing in mind the sample consisted of non and light-drinkers). This commonly manifested as resistance and disdain towards the drunken or aggressive behaviour of men. Interestingly, in Australia and the UK this contestation came from men more so than women. For example:

Like I said in our culture, Australia you know, that’s tradition. They’ve been raised to believe to the point where like sometimes it’s like, you’re not even really a man until you drink. Some families believe that, they actually teach their kids that. Which is crazy because it has nothing to do with being a man. It has nothing to do with - that doesn’t gain you anything so they shouldn’t be saying that basically. (Jake, 17yo, Australian)

On the other hand, while some women in Denmark and Sweden emphasised contesting traditional feminine drinking norms, it was not as common for men to contest traditional masculine drinking norms. Take this example, from a Danish participant, which is almost the opposite of Jake’s sentiment above: ‘Men drink stronger alcohol than girls. And this is what I experience, that people who are into being ‘a real man’ they drink what other men tend to drink, which is often beer’ (Peder, 18yo, Danish). When there were explicit challenges to masculine drinking norms in Denmark and Sweden, they were much more likely to come from women.

It is apparent that despite large declines in youth drinking in high-income countries, gender remains an important nature of performative work relating to drinking and non-drinking (both in terms of endorsing and resisting socially constructed ideas about gender). However, it was the ways intoxicated young women and men were described that provides greater insight into how gender might be implicated in these large decreases in youth drinking.

### Gendered expressions of others’ (non)drinking

While gender was implicit in many of the examples provided in the previous section, the most explicit references to gendered alcohol practices in data from Australia, Denmark and Sweden emerged when research participants described intoxicated men and women (see also Bogren, 2020; MacLean et al., 2020; Wilkinson & Wilkinson, 2020). Descriptions of drinkers were not always negative, with intoxicated men and women often talked about in positive (e.g., funny, spontaneous) ways. For example:

The last time I saw drunk women together, it just made them a lot more clingy to each other and they were all dancing and laughing and squealing – like to me that’s a lot more of the vibe I prefer. It’s about them having a nice time together. (Jasmine, 16yo, Australian)

However, the more common form of describing drinkers in Australia, Denmark and Sweden was to negatively describe intoxicated women and (particularly congregations of) intoxicated men. For example, intoxicated Australian, Danish and Swedish women were commonly described using terms such as ‘emotional’, ‘hysterical’ and ‘childish’. Both women and men used this language to describe women, but it was particularly notable among women. Consider the gendered language in the following quotes:

Girls tend to be more emotional, like breaking up and starting to cry or laugh hysterically, more emotions so. (Anna, 19yo, Swedish)

Loud, more attention-seeking, messy. They just don’t care what they’re doing, bitchy. They get pretty bitchy most of the time. (Sharna, 18yo, Australian)

Then they [drunk females] start to cry and stuff like that. Their boyfriend broke up, like two years ago, and then they… And I just don’t have the patience to deal with it, I just don’t. (Ann-Sofie, 19yo, Danish)

In the Danish data, it was particularly noticeable for women especially, but also men, to position intoxicated women as ‘too much’. While men were also discussed in negative ways, they were commonly positioned as less complicated and less annoying in their intoxication, and often more preferable drinking company. It was common for intoxicated women to be described in ways that drew on stereotypical notions of gendered traits in all countries. However, it was less common for female participants in Australia to engage with the concept of ‘inappropriate femininities’. Where this did occur in a few cases, it tended to come from men. For example:

I think there’s a level of embarrassment associated with a female getting drunk … I would rather my girlfriend not drink, unless… at this age, I would rather her not drink … Because I wouldn’t like the sight of it. (George, 17yo, Australian)

In the Nordic countries, while many women pushed back against the notion of appropriate femininities, celebrated being feminist-active, and boasted about ‘being one of the boys’ (e.g., drinking beer and watching sport with men), many women were also critical of young women who displayed what they considered to be inappropriate femininities:

I often see girls wearing too little clothes in the nightlife. Dancing, and then some guy comes up, and then they dance with him for a while and they get some free drinks. And then all of a sudden, they are not interested anymore. I think it’s a bit cheap and slutty. (Rikke, 18yo, Danish)

These somewhat paradoxical findings demonstrate the continued presence of traditional gender norms, alongside increasing resistance to these norms. Sometimes these opposing views came from different members of the sample, but it was also common for the same participant to both endorse and challenge traditional gender norms at various points in their interview.

Intoxicated men were most often negatively stereotyped when exhibiting ‘toxic’ forms of masculinity. In Australia this typically came from other men, whereas in Denmark and Sweden, this was mostly expressed from women. Consider the examples below where Luke and Sam critically questioned ‘jock-style’ forms of masculine expression:

I think the last time I saw that I was at the cricket. It might just be because they were at the cricket. But a bunch of drunk dudes together just looked a bit idiotic. I don’t know the right way to describe it without sounding rude. But if you know what I mean, sort of like a meat headed, bone headed, obnoxious I’d say. (Luke, 19yo, Australian)

I feel like it’s something they think is really manly or something that’s looked as being really manly and you’re like a big unit or a big dog or whatever you want to call it - like they’ve got some big presence in this social thing if they can get so pissed that they just don’t know what they’re doing. They do all this stupid stuff – they become like a hero for it. (Sam, 17yo, Australian)

While men in Denmark and Sweden were less likely to criticise groups of intoxicated men, many women did. For example, Lovisa (18yo, Swedish) used a word we have translated as ‘macho’ to describe in-toxicated men (i.e., masculine in an overly aggressive way). Australian women used words such as ‘angry’ and ‘violent’ to describe intoxicated men. Some women in Australia, Denmark and Sweden also described intoxicated men in ways that alluded to feeling unsafe around them due to the potential for predatory sexual behaviour; common terms used were translated as ‘creepy’, ‘weird’, ‘sleazy’ and ‘seedy’. Consider this comment from Ann Sofie: ‘Those guys who approach you and they are disgustingly drunk and lick you on the neck, it is so gross … They are so intrusive. Go away’ (Danish, 19yo). In these three countries women recounted many stories of unwanted physical contact from men, that was more ‘sexual’ than ‘romantic’ (Saga, 15yo, Swedish).

We did not identify obvious forms of gendered stereotyping regarding non or light drinking, with the exception that in Australia, non-drinking men were often described in ways that emphasised their healthy and muscular bodies, whereas in Sweden, it was women who were more often described as healthier, or less likely to drink heavily due to being more health and image-conscious.

There was much less gendered stigmatisation evident in the data from the UK. On the other hand, classed-based forms of stigmatisation were a recurring theme in interviews from the UK (see also Bogren, 2020), but these did not appear so much in the Australian and Swedish data. We develop this theme in the next section.

### Moderators or contingencies concerning gender and drinking

As with much theorising of gender and drinking, in all four datasets we interpreted ways that drinking practices or gendered stereotyping appeared to be moderated or contingently shaped by other factors, particularly class and cultural background (see also Bogren, 2020 ; Demant & Törrönen, 2011; Herold et al., 2020; MacLean et al., 2018; Törrönen & Maunu, 2007; Törrönen et al., 2017 Törrönen & Roumeliotis, 2014). Class as a moderator was noticeable in Australia, Denmark and the UK but less so in Sweden, where differences in drinking were related more to gender than to class, as noted previously: ‘gendered distinctions over-ride the socioeconomic distinctions’ (Törrönen et al., 2021). In the UK, however, class distinctions were observed to more commonly override gendered distinctions. This occurred in a few different ways, for example, through the use of classist stereotypes to describe drinking in public (particularly parks). Terms such as ‘scrotey’ (a derogatory term for a person, usually a man, who is deemed to be worthless, obnoxious or contemptible) and ‘chav’ (a highly classed stereotype of a young person who engages in aggressive, loutish behaviour) (Tyler, 2008) were used to describe and stigmatise intoxicated young men from rural or deprived areas who consumed alcohol in parks:

I’m not going to go out and drink in the park … It’s just not something you do is it, it’s a bit – what’s the word – I’m trying to think of a word that I can say on tape. Scrotey. … Deserving of an ASBO [antisocial behaviour order]. (Luke, 17yo, British)

Class-related forms of stigmatisation also occurred through the formulation of young people who drink heavily as low achievers and immature, such as in the following example:

There’s a correlation between kind of the bad kids, if you want to put it, and like drinking and drugs and all that … we’re not all model students, but we do well, we do good in the school … I’ve never seen [smart boys] going on a sesh or going drinking … you couldn’t imagine it and I think there’s [a] correlation between a bit of class and dignity and intelligence and then drinking. (Attaf, 16yo, British)

These constructions were noticeably more common from individuals interviewed from rural or deprived areas, perhaps due to being socially closer to the people they were stereotyping and therefore more actively working to distance themselves from these stereotypes, and/or alternatively, that middle class participants felt less comfortable adopting these stereotypes.

In Denmark, masculinity and class intersected in interesting ways, but not always consistently. For example, one participant noted that he felt he had to drink in ways that might traditionally be couched as masculine when around friends in vocational training, but could resist such performances when with high school friends:

If you are around people in vocational training, mostly carpenters, then it’s best to drink beer. Because they care a lot about being loud and manly, or whatever … masculine traits which apparently come with drinking beer. Whereas here, in high school (for trades) we have a tendency of being a bit more self-concerned. Feminine, perhaps. There’s a lot of stereotypes going on, but that is my experience. More people are saying ‘that’s what I choose to do, I choose to drink cider’. And that’s the attitude here. So when I drink with my friends from high school I can easily drink cider. (Nickolas, 18yo, Danish)

This quote hints at class-based constructions of gender and drinking, but also emphasises the importance of context as a moderator of gendered expression. Also in Denmark, it was common for participants to note that money (rather than social class per se) often served as (gendered) social capital in drinking environments. This applied to impressing the opposite sex, with one participant noting that ‘it’s cooler to buy Belvedere than Smirnoff because it looks like you have more money, then. You can show off a little bit, and then the girls usually come over’ (Peder, 18yo, Danish). It also applied to being a ‘king’ among friends:

Then he gets up with his credit card and almost proudly walks to the bar and buys the damn bottle. And when he returns to the table and puts it there, he’s just king, you know. In that situation, those who buys (pays for) the bottles, they are so cool in the eyes of the other guys. Cause they can afford it. (Camilla, 19yo, Danish)

These representations suggest that in the context of alcohol use, masculinity in Denmark is celebrated through demonstrations of material possession. In Australia, where interviewees were non and light drinkers, young men from middle class areas gave a stronger impression of holding hegemonic masculine values and were more likely to emphasise the desire to excel and/or be currently excelling in sport or academics; whereas young non-drinking men from working class areas were less likely to express masculinity through competition, and had a much more diverse range of reasons for drinking lightly or non-drinking. We identified few examples where class and femininities clearly intersected, which is perhaps surprising given the voluminous literature on this topic (e.g., Hutton, 2020; Jackson & Tinker, 2007; Nicholls, 2019 Vaadal & Ravn, 2021), but might potentially be explained by the younger age of our samples or the high proportion of non- or light-drinkers in the samples.

The other primary moderator evident in our samples was race and religion, which clearly intersected with gender in Sweden, Australia and UK (although not so much in Denmark, potentially due to less ethnic diversity in the sample). In all countries, participants from ethnic minority backgrounds (particularly Asian and African) were more likely to be abstainers or light drinkers. In the UK and Australia, those who identified as Muslim often cited their religion as the reason for non-drinking, while some African participants identified their parents and their parenting styles as the reason. There were gendered elements to this, for example:

Our culture [South Sudanese], they prohibit drinking and smoking, any of those type of behaviours … There’s a certain age where that’s stops being so strict. They [our parents] just don’t want us to try too early before our minds are fully developed. So, when your mind is fully developed, you’re a man, you’re doing well in life, you just occasionally drink with your friends, the boys, whatever, that’s when they’ll be like, all right that’s fair enough, but watch that. Don’t overdo it, don’t overstep your boundaries, don’t like push the limit, just relax. (Adam, 17yo, Australian)

Here, Adam clearly positioned himself as a boy, not yet ‘a man’ with neuro-developmental capability of expressing self-control over an intoxicating substance; a message that had come from views rooted in his cultural background and endorsed by his family. We also noted that young White-Australian and White-Swedish participants were more likely to endorse traditionally masculine and feminine drinking norms (implicitly) than non-White Australians and non-White Swedish participants. In Australia, White-Australians and those from Anglophone European countries were also more likely to participate in explicit negative same-sex gendered stereotyping or stigmatising of other men and women (i.e., Asian and African participants were much less likely to negatively stereotype drinkers). This was different in Sweden where participants with Middle Eastern backgrounds were more likely to negatively describe drinking (rather than drinkers per se).

These findings reiterate the importance of attention to the moderators and contingencies of gendered drinking practices, both in terms of own (non)drinking and the positioning of others’ (non)drinking. In the discussion to follow, we further interpret our findings with a particular focus on whether and how they shed light on the role of gender in the context of declining drinking practices among young people.

## Discussion

In our examination of the ways in which gender was expressed in relation to participants’ own drinking, we identified that, consistent with previous analyses (e.g., Frank et al., 2020; Measham, 2002; Nicholls, 2020), drinking practices enabled the opportunity for stereotypical gendered expressions and performances (such as same-sex bonding), as well as opportunities for engagement with, and demonstrations of, hegemonic masculinities and ‘appropriate’ femininities. There were also many examples where traditional displays of masculinity and femininity through drinking practices were contested (see also Bogren, 2020; Measham, 2002; Törrönen & Roumeliotis, 2014), for example, through the rejection of what participants perceived to be the dominant norms or expectations around gender self-presentation and behaviour. These findings, while important to note, do not obviously inform how gender might be implicated in declines in youth drinking, which we turn to for the remainder of the discussion.

The negative stereotyping of intoxicated women and men was a prominent theme in data from Australia, Denmark and Sweden, and we noted that the way young intoxicated men and women were negatively described was heavily gendered. Women were most often described as ‘emotional’ and ‘moody’ when drunk, sometimes they were referred to as ‘bitchy’ or ‘childish’, and in Denmark in particular they were negatively described relating to sexuality (both in terms of appearance and behaviour). While women have often been criticised by other women – and also men – for their appearance and sexual conduct while intoxicated (Griffin et al., 2013; MacLean et al., 2018), their consistent gendered descriptions as hysterical, irrational, juvenile, and mean was prominent and remarkably consistent across three of the four samples, and from both drinkers and non-drinkers. It is important to note that women’s drunkenness was also positively described, with many participants preferring the company of intoxicated women over men (this was most notable in Australia). However, it was equally, if not more common, for women to be couched in ways that indicated that their company was ‘too much’ and they should be avoided when drunk (most notably in Denmark).

Intoxicated men too, were negatively described and again the language was highly gendered to describe either the threat of their unpredictable or aggressive behaviour, or their predatory or unsanitary behaviour. In Australia, this was a particularly strong finding; with men most often engaging in these stigmatising practices and usually applying these descriptors to congregations of intoxicated men. In Denmark and Sweden, these descriptions more commonly came from women, with men more likely to positively describe groups of intoxicated men in these countries. The valorisation of controlled drinking, and disparagement of inebriation, has been a feature in the declining youth drinking literature (e.g., Caluzzi, MacLean, et al., 2021; Caluzzi et al., 2020); however, the gendered nature of this stereotyping is illuminating. While displays of sexual behaviour in drinking settings have long been the subject of scrutiny (for both women and men), we identified a range of gendered slurs, with women described as annoying, immature and pathetic and men as idiotic, threatening and disgusting. This may provide insight into gender expectations more generally; for example, an intolerance toward outdated constructions of gender that emphasise weakness for women and strength for men. Young women and men expressing disdain for these traits suggests disapproval of the intoxicated performance of objectionable and regressive gendered norms. It is important to note that describing intoxicated women and men using pejorative gendered language is not a new phenomenon (e.g., Jackson & Tinker, 2007; Peralta & Cruz, 2005) and that we do not have similar or comparable baseline data with which to track changes over time. However, with the exception of the UK study where class was a stronger theme, we were struck by the persistence of these findings. Quantitative analyses of gendered drinking norms and performances among young people over time would be a worthy avenue of investigation should appropriate data be available to assess whether these have changed over time.

A second point of consideration that may shed light on the role of gender in declining youth drinking is the way masculinities and femininities were expressed through non and light drinking choices. In the countries that included non-drinkers, particularly in Australia and Sweden, gender was apparent in performative work related to the construction of non-drinking identities, particularly related to health, sport, academic success and religion. This has been observed and covered in rich detail for Swedish and Danish participants (Frank et al., 2020; Törrönen et al., 2019; Törrönen et al., 2020). We did note a subtle difference between Australian and Swedish participants on this issue, with men in Sweden often focusing on sport and gaming as ways of performing non-drinking masculinities and women on healthy bodies and wellness. In Australia, young men were more likely to draw on healthy bodies, along with academic success, as ways of performing masculinity but women drew on a larger variety of non-drinking femininities (such as artistic and religious pursuits), and less on health and wellness. It may be that performing masculinity remains ultimately related to competition (at least among those with stronger hegemonic masculinity ideals), be it through sport, physical prowess, gaming or academic achievement, but that femininities are more individual and personalised, permitting more variety.

We also noted that light and moderate drinking practices offered up ways of ‘doing gender’, particularly through engagement with notions of maturity and neoliberal responsibilisation. It was through light and moderate drinking performances that participants valiantly emphasised their aptitude for discipline and control. They positioned themselves as more ‘grown up’ than their drinking peers, and more mature than previous generations at the same age. This supports the early-maturation suggestion put forward by Törrönen and colleagues that young people today may be more responsible, reflective and adult-like than previous generations (Törrönen et al., 2019). However, our findings further this by showing engagement with these maturities is gendered, with participants drawing on stereotypical traits of mature men (‘being a man’, handling oneself and protecting others), and women (knowing their limits, protecting oneself and keeping oneself ‘tidy’).

We noted some subtle but important differences in the way gender was performed and described through drinking between countries. For example, we noted strong resistance to performances of toxic drinking masculinities across the countries; however, this tended to come from men in Australia and the UK, and women in Denmark and Sweden. In Australia and the UK it was common for young men to describe avoiding intoxicated men who could become aggressive and violent, especially when in groups. Normative masculinity through drinking was often formulated as maturity, and being able to control one’s actions, even when drinking more than moderately. In Sweden and Denmark, however, men continued to valorise drinking as a way to perform masculinity while drinking. There are two potential explanations for this cross-cultural difference; the first is that intoxicated young men in Australia and the UK may be more likely to behave in ways constructed as intimidatingly masculine when intoxicated and so men who drink lightly or not at all may have formed negative views on this. On the other hand, in the Nordic countries, men’s heavy drinking may be less aggressive or unpredictable; that is, there may be more room to perform masculinity when drinking without engaging in these types of practices. Another possibility is that young men in Sweden and Denmark are showing less inclination to resist the reshaping of drinking masculinities. There may have been a stronger push from men in Australia and the UK to reshape or restructure gender power imbalances (over and above – or to catch up to – gender relations in Nordic countries), which is being picked up by participants in the way they both describe themselves, and the way they describe other men.

Opposingly, women in Sweden and Denmark were vocal in their denigration of intoxicated young men. Women in these countries showed significant resistance to stereotypical drinking masculinities. They were also more likely to engage in the stigmatisation of intoxicated women, but this was often paradoxical – with those who endorsed and violated ‘appropriate femininities’ often criticised by different (and sometimes the same) participants. Sweden and Denmark are among the most progressive in the world with regards to policies on gender equality and so this is an important ambivalence. It appears as though young Nordic women are both more progressive in reshaping femininities of drinking but are at the same time actively resisting this process. This may signal a culture in transition; indeed, it appears to reflect different paces of change across and within countries, speaking to the importance of positioning these as fluctuating and evolving, rather than static, societies.

We also noted some gender differences with regards to how class and cultural background shaped drinking practices. In the UK for example, intoxicated young men from deprived and rural areas were spoken about in derogatory ways that were highly classed or placed. The intersection between social class and masculinities of drinking was also noticeable in Denmark, with stereotypical hegemonic drinking masculinities endorsed in trade and vocational arenas, as well as in bars among those with greater access to financial resources. In Australia, on the other hand, young men from lower socio-economic areas and non-white cultural groups were far more progressive in resisting toxic masculinities and drinking, and white-Australians from middle class areas were far more hegemonic in their masculine ideals (a finding also evident in Sweden). These findings reiterate those established elsewhere that gendered drinking practices and gendered stereotyping is contingently shaped by class and cultural background (Bogren, 2020; Demant & Törrönen, 2011; Herold et al., 2020; Törrönen & Maunu, 2007; Törrönen et al., 2017; Törrönen & Roumeliotis, 2014), and highlights the need for research on gender to not only look across cultures, but at the moderators and nuances within cultures.

We would like to make note of several limitations to our analysis. First and foremost, in this article we adopted a binary construction of gender, which is problematic and reflects a general over-emphasis on binary gender categories in alcohol research. While none of our participants identified as non-binary (potentially due to their young age), assuming that gender is a fixed category is, and will become increasingly, problematic as gender fluidity becomes more normative (Hunt & Antin, 2019). This needs to be addressed in a meaningful way in future research efforts, particularly in quantitative studies where categories are fixed and nuance is more difficult to elaborate. This does not mean that femininity and masculinity are not useful constructs for investigation; indeed, we have highlighted the ways (non)drinking practices offer important opportunities to resist or contest traditional constructions of gender and this can illuminate the importance of shifts in broader social processes.

It is also important to note that our analysis draws on four individually collected studies; which involved varied designs, sampling focus, and research questions. For example, much of the UK data were drawn from group interviews conducted in educational settings which may have influenced how freely some participants felt in speaking about ‘unacceptable’ forms of femininity and masculinity relative to participants in the other studies. Data were analysed by individuals in each country and it is important to emphasise the reflexivity inherent in our personal constructions of gender and how this undoubtedly shaped our reading of the data. This is further complicated, but also made more interesting, by our ideas and understandings of the different countries that we live and work in.

## Conclusion

Our analysis of qualitative data with drinkers and non-drinkers from four high income countries provides some clues about the role of gender in declining youth drinking. Heavy drinkers and states of intoxication (particularly those resulting in loss of control) were described in highly gendered pejorative terms in three of the four countries. This suggests that, for some young people, intoxication is associated with facilitating or enabling constructions or performances of gender that are regressive and not permittable or appropriate displays of gender performance for young men and women. Whether and how this has changed over time requires further investigation through longitudinal methods. Resisting stereotypical displays of gender while drinking, and contesting traditional gender norms, also seems to offer an opportunity for expressing gendered drinking identities through performances of emotional maturity and physical orderliness. Non- and light-drinking practices also offer a range of opportunities for expressing ‘appropriate’ or desirable feminine and masculine positions that fit within these boundaries. It is here we might identify opportunities for harm reduction policies; that is, supporting ways of ‘doing gender’ with friends in social environments without heavy drinking. Importantly, our findings specify how gender contributes in different ways to the place of drinking for young people between and within different cultural contexts, highlighting the importance of investigating and understanding these contingencies and their continually evolving nature.

## Figures and Tables

**Table 1:** Sample demographics.

Australian sample (2018)	n=50 (%)
*Gender* ^^^	
Woman	26 (52)
Man	24 (48)
*Age*	
16	10 (20)
17	29 (58)
18	6 (12)
19	5 (10)
*Drinking status*	
Abstainer	25 (50)
Light drinker	25 (50)
*Place*	
Inner-metropolitan Melbourne	16 (32)
Outer-metropolitan Melbourne	29 (58)
Regional Victoria	5 (10)
*Socioeconomic status* ^a^	
Working class	26 (52)
Middle class	24 (48)
*Education status*	
Currently in high school	39 (78)
Currently at university	11 (22)
*Cultural background* ^b^	
Australian born parents	28 (56)
One or both parents born elsewhere	8 (16)
Born elsewhere	14 (28)
**Danish sample (2015-16)**	**N=35 (%)**
*Gender* ^^^	
Woman	14 (40)
Man	21 (60)
*Age*	
18	21 (60
19	14 (40)
*Drinking status*	
No last month heavy episodic drinking	5 (14)
Heavy episodic drinking 1-2 times in past month	13 (37)
Heavy episodic drinking 3+ times in past month	17 (49)
*Place* ^c^	
Large city (Copenhagen, Aarhus)	4 (11)
Large provincial town	10 (29)
Small provincial town, village, rural area	21 (60)
*Education status*	
Currently in or just finished high school	27 (77)
Vocational training	5 (14)
Other	3 (9)
*Cultural background*	
Born in Denmark with Danish-born parents	32 (91)
Born in Denmark with one parent born elsewhere	2 (6)
Born elsewhere	1 (3)
**Swedish sample (2017)**	**n=56 (%)**
*Gender* ^^^	
Woman	32 (57)
Man	24 (53)
*Age groups*	
15-17	26 (46)
18-19	30 (54)
*Drinking status*	
Non-drinkers	28 (50)
Moderate drinkers	15 (27)
Heavy episodic drinkers	13 (23)
*Place* ^d^	
Large city	8 (14)
Middle-sized town	22 (39)
Smaller town	26 (46)
*Socioeconomic status* ^e^	
Lower	19 (44)
Middle	26 (46)
Higher	11 (20)
*Cultural background*	
Swedish born parents	36 (64)
One or both parents born elsewhere	15 (27)
Born elsewhere	5 (20)
**UK sample (2019)**	**n=53 (%)**
*Gender* ^^^	
Woman	28 (53)
Man	25 (47)
*Age*	
16	20 (38)
17	14 (26)
18	14 (26)
19	5 (9)
*Drinking status*	
Abstainer	12 (23)
Light drinker	10 (19)
Drinker	31 (58)
*Place/socio-economic status* ^f, g^	
Affluent school	14 (26)
Deprived school	10 (19)
Rural school	16 (30)
Further education (FE) college	6 (11)
University	7 (13)
*Education status*	
Currently in secondary school	40 (76)
Currently in FE college	6 (11)
Currently at university	7 (13)
*Ethnic background*	
White British	35 (66)
White Other	4 (8)
British South Asian	5 (9)
Mixed White-South Asian	4 (8)
Black African	3 (5)
Black British	2 (4)

^ No participants in the age groups included in our analysis identified as transgender or non-binary.

a Socio-economic status of Australian participants was rudimentarily categorised based on suburb (socio-economic indexes for areas (SEIFA), Australian Bureau of Statistics, 2018) and in a few cases, re-classified based on participant responses (e.g. where it was clear participants lived in a wealthier area but whose parents were struggling financially).

b Of Australian participants who spoke a language other than English at home (i.e., first- or second-generation immigrants) fifteen had Asian parents (Indonesian, Korean, Chinese, Indian, Sri Lankan, Malaysian, Vietnamese, and Cambodian), five had European parents (Greek, Turkish, German, Dutch, Russian) and two had African parents (Sudanese and Ethiopian).

c No information about socio-economic status or parental income was collected in Denmark.

d Based on number of Swedish inhabitants in the municipality of residence. Large city ≥ 500,000, Middle town 50,000–499,999, Small town < 50,000.

e Based on categorization of share of adult Swedish population with long-standing social welfare, average income of population, and ill-health rate of the municipality of residence.

f The relative affluence of UK schools was established using data on the proportion of pupils eligible for free school meals.

g Relative rurality in the UK was determined using distance from the city centre and local area knowledge. Rural schools were comparable in their levels of deprivation to the urban deprived school.
